# Automated segmentation of complex patterns in biological tissues: Lessons from stingray tessellated cartilage

**DOI:** 10.1371/journal.pone.0188018

**Published:** 2017-12-13

**Authors:** David Knötel, Ronald Seidel, Steffen Prohaska, Mason N. Dean, Daniel Baum

**Affiliations:** 1 Zuse Institute Berlin, Dept. of Visual Data Analysis, Berlin, Germany; 2 Max Planck Institute of Colloids and Interfaces, Dept. of Biomaterials, Potsdam-Golm, Germany; Chongqing University, CHINA

## Abstract

**Introduction:**

Many biological structures show recurring tiling patterns on one structural level or the other. Current image acquisition techniques are able to resolve those tiling patterns to allow quantitative analyses. The resulting image data, however, may contain an enormous number of elements. This renders manual image analysis infeasible, in particular when statistical analysis is to be conducted, requiring a larger number of image data to be analyzed. As a consequence, the analysis process needs to be automated to a large degree. In this paper, we describe a multi-step image segmentation pipeline for the automated segmentation of the calcified cartilage into individual tesserae from computed tomography images of skeletal elements of stingrays.

**Methods:**

Besides applying state-of-the-art algorithms like anisotropic diffusion smoothing, local thresholding for foreground segmentation, distance map calculation, and hierarchical watershed, we exploit a graph-based representation for fast correction of the segmentation. In addition, we propose a new distance map that is computed only in the plane that locally best approximates the calcified cartilage. This distance map drastically improves the separation of individual tesserae. We apply our segmentation pipeline to hyomandibulae from three individuals of the round stingray *(Urobatis halleri)*, varying both in age and size.

**Results:**

Each of the hyomandibula datasets contains approximately 3000 tesserae. To evaluate the quality of the automated segmentation, four expert users manually generated ground truth segmentations of small parts of one hyomandibula. These ground truth segmentations allowed us to compare the segmentation quality w.r.t. individual tesserae. Additionally, to investigate the segmentation quality of whole skeletal elements, landmarks were manually placed on all tesserae and their positions were then compared to the segmented tesserae. With the proposed segmentation pipeline, we sped up the processing of a single skeletal element from days or weeks to a few hours.

## Introduction

Over the past decade, image acquisition techniques like computed tomography have developed to allow visualization of biological structures at a level of detail previously possible only in industrial or synchrotron scanners. Moreover, computed tomography has become affordable and efficient enough to permit scanning of a large number of specimens in a short period of time, thus enabling quantitative statistical analyses beyond qualitative descriptions. As a result of these advancements, allowing ever-faster generation of ever-larger amounts of data, there is a growing need for automation of image analysis tasks. However, even though most computed tomography-based research on geometric structures in biology (e.g. individual morphological features) demands image segmentation—the process of assigning a class label to voxels of the image in order to digitally isolate them—no general purpose image segmentation method exists for similar types of data. Although there are common strategies that can be routinely applied, often a single algorithm is not sufficient to solve a specific image segmentation task. Instead, a sequence of image analysis methods including image filters, binary segmentation, and object separation is needed to achieve the desired results and commonly, such a pipeline needs to be adjusted to the specific kind of data.

**Fig 1:**
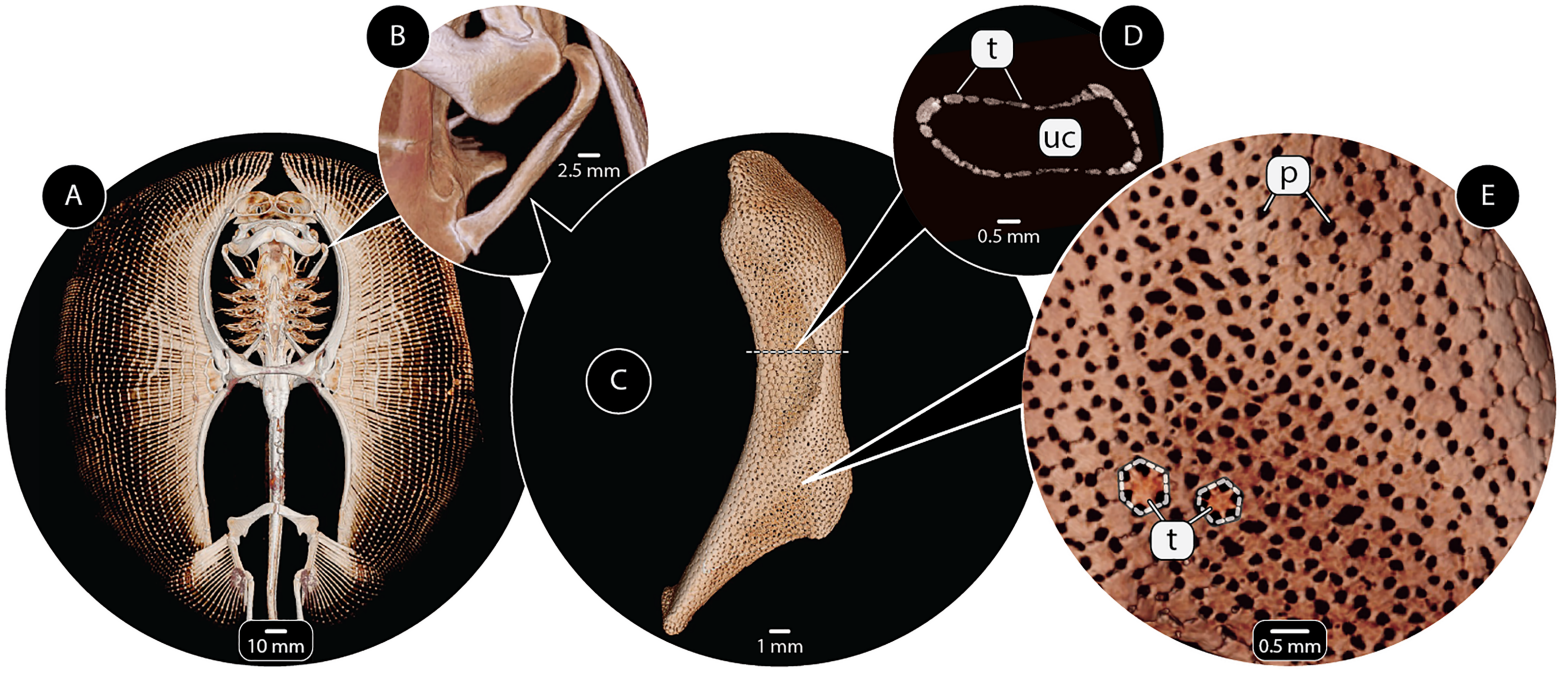
Tessellated cartilage of the stingray *Urobatis*, at multiple levels of structural organization. (A) The skeleton is visible in CT scans due to the mineralization of the cartilage. (B,C) The hyomandibula, a skeletal element connecting cranium and jaws. (D) Transverse section of the hyomandibula—the outer layer of mineralized, tessellated cartilage (tesserae, t) is visible, surrounding an inner core of unmineralized, radiolucent cartilage (uc). (E) Surface view of the hyomandibula; note the variation in the shape of tesserae and the size of pores (p). Tesserae can be demarcated by connecting the pores between adjacent tesserae. Specimens: (A) *Urobatis concentricus* (USNM87539), medical CT; (B-E) *Urobatis halleri*, μCT.

In this paper, we present a pipeline for the semi-automatic segmentation and geometric reconstruction of repeating sub-units in volumetric data, a common structural motif in biology [1]. The segmentation workflow can be tailored for different data types. For the development of our pipeline, however, we investigate the skeletons of sharks and rays, which pose particular challenges for segmentation. Shark and ray skeletons are made of unmineralized cartilage wrapped in an outer layer of mineralized polygonal blocks called tesserae (Fig 1) [2–5]. Although this ‘tessellation’ has been known for over a century as a defining feature of all shark and ray skeletons, the complex 3-dimensional morphologies and arrangements of tesserae (Fig 1E) and their small size (typically less than 500 μm in all dimensions) have limited any efforts at quantification of tesseral morphologies and networks. Tesserae, however, can be beautifully resolved in micro-computed tomography (e.g. [5, 6]), making them a useful system to test tools for automatic or semi-automatic segmentation of challenging biological data.

The automation of segmentation depends on the ability to reliably isolate objects of interest (foreground) from the background, as well as from one another. In many segmentation workflows, this is accomplished by exploiting gray value differences between foreground and background (e.g. via thresholding or watershed transform on an edge image) and/or by measuring the distance of foreground to background voxels (e.g. via distance transforms) to detect object boundaries. Conventional pipelines employing these techniques, however, are not effective for this system due to the complex ultrastructure of tesserae.

**Fig 2:**
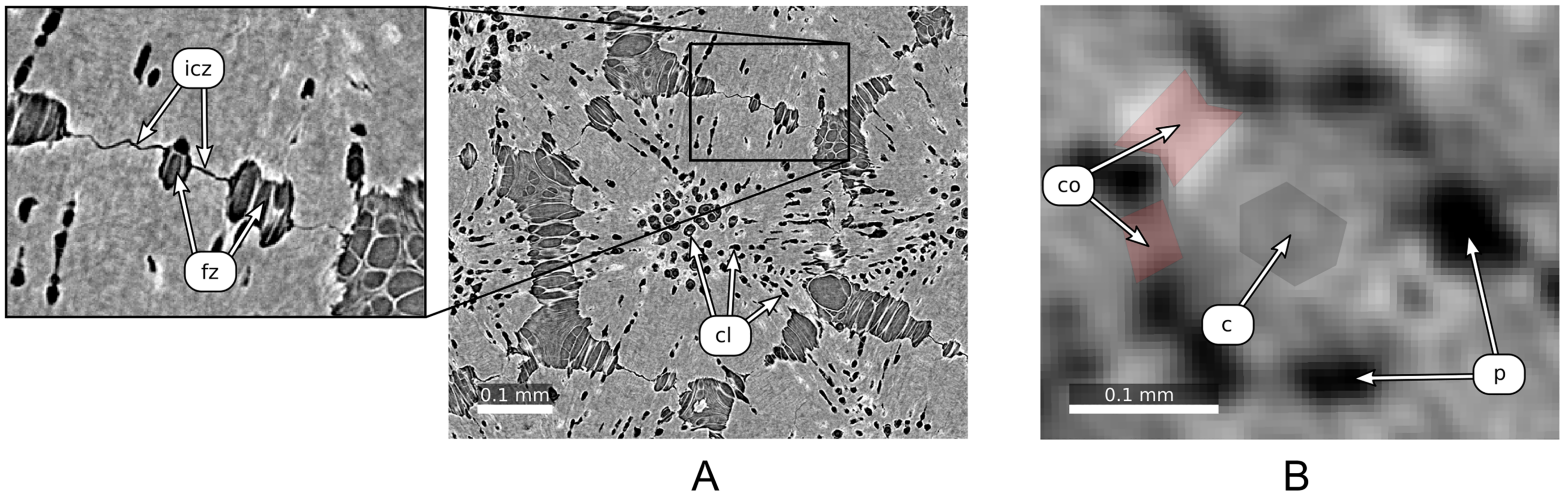
μCT images of tesserae acquired with different resolutions. Images (A) and (B) show a single tessera surrounded by neighboring tesserae. (A) Synchrotron μCT image with voxel size 0.678 μm. In the center of the tessera, many cell lacunae (cl) are visible. The close-up shows an inter-tesseral joint consisting of inter-tesseral contact zones (icz) with direct contact between adjacent tesserae and fibrous zones (fz) without direct contact. (B) Voxel size: 4.89 μm. This image shows the native resolution of the μCT scans used in this paper, before being downsampled for analysis to 9.78 μm (see ‘Input Data’ below). Note that the inter-tesseral contact zones and small fibrous zones cannot be seen since the resolution is not high enough. Hence we use the following terminology throughout this paper: Inter-tesseral *connection (co)* for the entire connection between tesserae, including both contact and fibrous zones and appearing as areas of high intensity (high gray values) between tesserae, unmineralized *pores (p)* for areas of low intensity between tesserae, and *tessera center* (c) for the region around the center of a tessera.

First, tesserae are not easily separated by gray values, because the gaps between them (low gray value inter-tesseral joints; see Fig 2 for our terminology throughout this paper) are smaller (less than 2 μm) than the voxel size of our scans. As a result, these joints often disappear in μCT scans (Fig 2B) and tesserae appear joined, although higher resolution techniques (e.g. synchrotron μCT or scanning electron microscopy) show that this is not the case. Such high-resolution techniques, however, sacrifice field of view for resolution and can only capture a small number of tesserae, not a whole skeletal element with thousands of tesserae. Hence, for the segmentation task described in this paper, the scans have resolutions comparable to that shown in Fig 2B, to allow large-scale segmentations over entire pieces of the skeleton.

To avoid problems that some materials pose for gray value-based segmentations, several works have used combinations of the watershed transform [7] and a distance transform [8] to segment objects in contact based on their shape: soil particles [9–13] and glass beads [14, 15], but also biological objects, such as clustered nuclei [16] and neuron somata [17]. Some aspects of tesserae, however, complicate their segmentation via conventional shape-based methods that use a 3D distance transform to segment objects according to their geometry. Tesserae are relatively thin, and therefore, the width of the inter-tesseral connections (i.e. the distance between two pores; Fig 2B) may be larger than the height of the tesserae. That is, the third dimension (here, the height) might be smaller than the other two dimensions and is therefore inadequate for object separation (i.e. the other two dimensions should be used). Furthermore, tesserae are perforated by many small cavities (cell lacunae) [5, 18] (Fig 2A) that can further complicate the designation of foreground and background. We have found that these structural features of tesserae, in conventional segmentation workflows (e.g. when a hierarchical watershed transform is applied to a 3D distance map), often result in tesserae being segmented into several pieces (oversegmented) rather than being separated from each other.

### Overview of the segmentation pipeline

**Fig 3:**
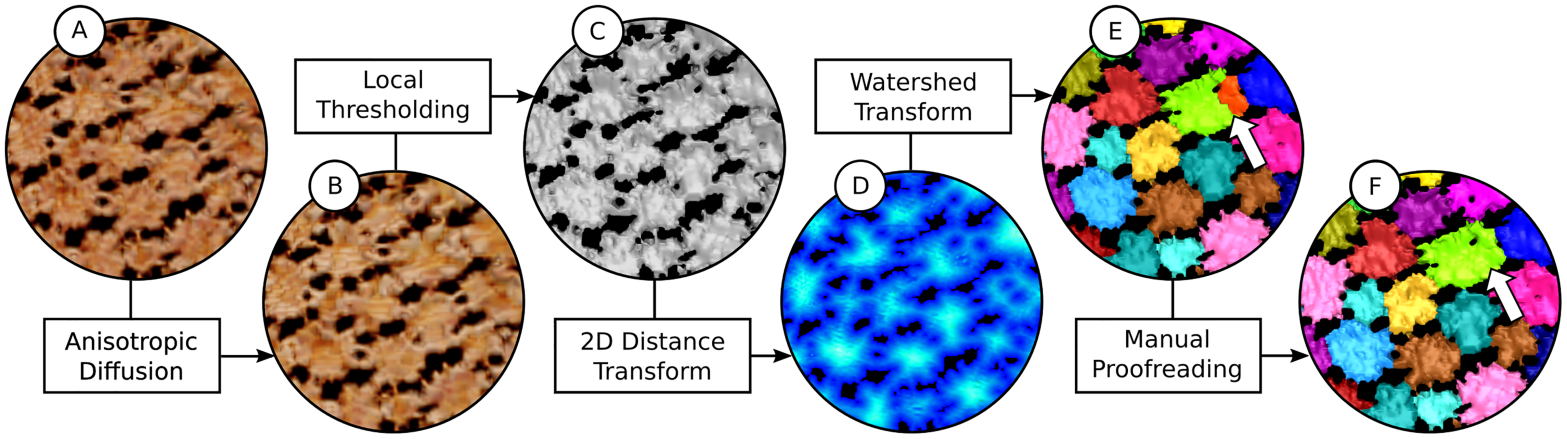
Overview of the segmentation pipeline. (A) Volume rendering of input μCT image; (B) Preprocessing result: volume rendering of input image smoothed with anisotropic diffusion to maintain edges. Differences to (A) are not visible here, but smoothing the image improves the segmentation significantly; (C) Surface representation of foreground segmentation, now tesserae are separated from the background using local thresholding; (D) 2D distance map measuring distances to pores between tesserae; (E) Segmentation result after applying hierarchical watershed transform; (F) Postprocessing result: segmentation after manual error corrections, the arrows in (E) and (F) highlight a segmentation error due to a hole inside a tessera which is corrected by merging two segments.

We circumvent these problems by combining traditional and modified segmentation tools in a five-stage pipeline (Fig 3), which takes into account the specific morphological and ultrastructural aspects of tesserae discussed above. In particular, we implement a specialized 2D distance transform, which addresses the segmentation issues caused by the flatness of tesserae, limiting the measurement of voxel distances to two dimensions, thereby avoiding issues traditional 3D distance maps may cause. The result is a high-quality segmentation of the mineralized layer of whole skeletal elements comprising several thousand tesserae. Compared to a fully manual segmentation, we speed up the processing of a single skeletal element from days or weeks to a few hours. The segmentation is performed using fast automatic algorithms, which can be followed by manual error corrections to enhance the segmentation result. The pipeline is modular and can be modified for the segmentation of other biological tissues. The individual steps are listed below.

1. *Anisotropic diffusion*: Remove intra-tesseral holes (cell lacunae) using anisotropic diffusion to avoid oversegmentation of tesserae.
2. *Local thresholding*: Apply local thresholding based on a region of interest around every voxel, in order to generate a binary foreground image of the tessellated layer. The local thresholding stage accounts for variations in mineral distribution across skeletal elements. Not all tesserae possess the same range of mineral densities [5]; the gray values of the mineralized cartilage therefore vary between different regions of the skeletal element, making a binary segmentation using global thresholding infeasible.
3. *2D distance transform*: Compute a 2D distance map on the binary foreground image, measuring the distances to adjacent pore spaces and transforming the binary image into a scalar field. This allows shape-based—rather than gray value-based—separation of tesserae in the following step.
4. *Hierarchical watershed transform*: Separate the tesserae by applying a hierarchical watershed transform to the 2D distance map. Since the interconnections between tesserae are narrower than the tesserae are wide (Fig 2B), and voxels near inter-tesseral joints are close to background voxels (within pores), the 2D distance map enables automatic separation of most tesserae from one another, even when no inter-tesseral joint space is visible.
5. *Manual proofreading*: Clean up segmentation results using custom tools for proofreading and interactive enhancement that allow easy correction of segmentation issues, such as over- and undersegmentation or inaccurate splits between adjacent tesserae. To aid with this, we create a graph representation of the tesseral network, where each tessera center is represented by a single node, linked to neighboring tesserae nodes by edges (connecting elements). This graph enables the detection of segmentation errors, while also allowing comfortable user interaction.

We describe each stage of the pipeline in detail in the following ‘Materials & Methods’ section, followed by a discussion of practical steps for implementation of the method in ‘Practical application and parameters’, and then a quantitative assessment of individual pipeline stages in ‘Evaluation’.

## Materials and methods

### Input data

We apply the segmentation pipeline to hyomandibulae (rod-like skeletal elements linking the jaw and cranium) harvested from specimens of round stingray *Urobatis halleri*, donated from another study [19]. Specimens were all sub-adults/adults and collected by beach seine from collection sites in San Diego and Seal Beach, California, USA. Hyomandibulae were mounted in clay, sealed in ethanol-humidified plastic tubes and scanned with a Skyscan 1172 desktop μCT scanner (Bruker μCT, Kontich, Belgium) in association with another study [5]. Scans for all samples were performed with voxel sizes of 4.89 μm at 59 source voltage and 167 μA source current, over 360° sample rotation. For our segmentations, the datasets were resampled to a voxel size of 9.78 μm to reduce the size of the images and speed up processing.

In all datasets, tesserae appear as flat, thin, 3D objects covering the surface of the hyomandibulae (Fig 1C–1E). The width of tesserae, in general, is larger than the width of their inter-tesseral connections (see Fig 2B). The workflow is tailored to tessellated cartilage, but in general will work for 3-dimensional (3D) gray-scale images containing objects that may be connected to each other with respect to the gray values but whose connections are smaller than the size of the objects in at least one dimension. The correct boundary between two objects has to be the thinnest part of their connection (thin according to the chosen dimension). As a further prerequisite for our input data, we require that the size of the objects in any dimension should be at least a few voxels, see Fig 2.

### Segmentation pipeline

In order to formally describe all steps of our image processing pipeline, we define a 3D image *I* as a scalar function $I:{\mathbb{R}}^{3}⊃\Omega \to \mathbb{R}$ of intensity values over a compact domain that is discretized via a regular grid *Ω*. Furthermore, if *x* is a grid node and *l* is a scalar value, then we denote by *B*(*x*, *l*) the set of all grid nodes in a cube with edge length *l* around center *x*.

The goal is to segment individual tesserae in the input image *I*. Tesserae are mineralized and the surrounding tissues are not. We exploit voxel intensities to separate tesserae voxels from background voxels and tesseral shape (their narrow connections and pores) to separate tesserae from one another.

#### Anisotropic diffusion

As a preprocessing step, we apply anisotropic diffusion [20] to effectively smooth the image and remove holes appearing inside tesserae. In contrast to the inter-tesseral pores that separate the tesserae from one another, the intra-tesseral holes (cell lacunae) are much smaller in size and have lower gray value differences compared to the surrounding mineralized material. Hence, anisotropic diffusion will preserve pores while smoothing away (most of) the intra-tesseral holes.

The diffusion equation is given as

$$\begin{array}{c} \frac{\partial I(x,y,z,t)}{\partial t}=\nabla \cdot \left(D(∥\nabla I\left(x,y,z,t\right)∥)\nabla I\left(x,y,z,t\right)\right) \end{array}$$

where *t* is the time of the diffusion process and *D* is a diffusion function controlling the diffusion process. We use a modified GPU-based version from Bernard et al. [21] where the diffusion coefficient assigned to each face between adjacent voxels is equal to 0 or 1 depending on the corresponding gray value difference. Therefore diffusion stops between neighbored voxels with an intensity difference larger than a user-defined threshold *T*_*s*_. We denote the smoothed image by *I*_*s*_.

#### Local thresholding

In the next step, we want to separate the mineralized cartilage comprising all tesserae, subsequently called foreground, from the unmineralized cartilage and the region outside the skeletal element, which we subsequently call background.

**Fig 4:**
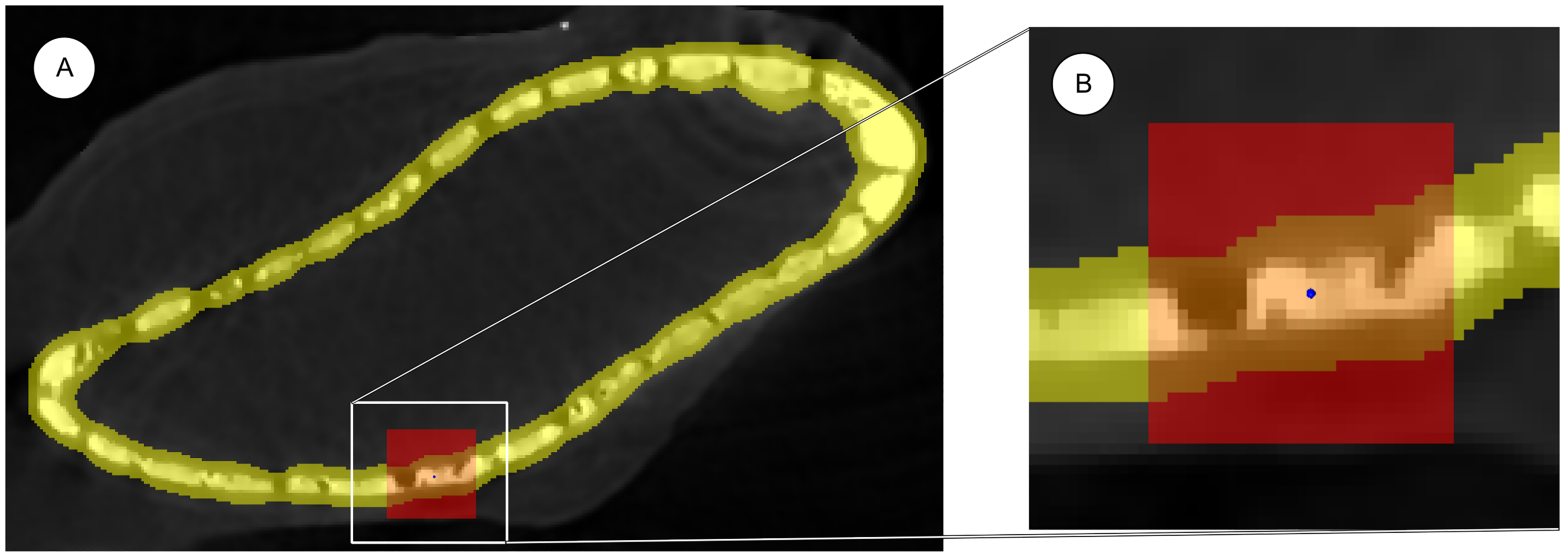
Neighborhood for local thresholding. (A) Slice through smoothed image *I*_*s*_ with *S* highlighted in yellow and *B*(*x*, *l*) in red, the blue point denotes the voxel *x*; (B) Close-up of box in (A) with *N*(*x*) ≔ *B*(*x*, *l*) ∩ *S* highlighted in orange.

Let *F* ⊂ *Ω* be the set of all foreground voxels. Tesserae voxels have larger intensity values than background voxels. But due to varying intensities across *I*_*s*_ (e.g. due to mineral density variation within tesserae), simple global thresholding fails. Therefore we use a local thresholding algorithm with

$$\begin{array}{c} x\in F⟺x\in S\wedge I_{s}\left(x\right)>\frac{T_{n}}{\left|N(x)\right|}\sum_{y\in N(x)}I_{s}\left(y\right)\;, \end{array}$$

where *N*(*x*) is the neighborhood around *x* defined as

$$\begin{array}{c} N(x)≔B(x,l)\cap S\;, \end{array}$$

and *S* is an area around the tesserae layer (see Fig 4). Finally, background voxels completely surrounded by foreground voxels are included into the foreground. So the value of *I*_*s*_ at position *x* is compared to the average value in a neighborhood; the ratio is controlled by threshold parameter *T*_*n*_. The neighborhood comprises all voxels in a cube *B*(*x*, *l*) around *x* that are also inside the user-defined area *S*. The region *S* is used to exclude large regions that obviously belong to the background. It prevents background voxels far away from the mineralized cartilage to be classified as foreground voxels. *S* is generated by region growing starting from a tolerant global thresholding. The edge length *l* has to be large enough to include at least some background voxels when centered at a tesserae voxel. It also needs to be large enough to include some tesserae voxels when centered at a voxel in *S* not belonging to the mineralized cartilage.

#### Two-dimensional distance map

The distance map should measure the distance of a voxel to the nearest porespace. Hence, we propose to utilize a two-dimensional (2D) distance map that, for each foreground voxel, restricts the distance computation to the plane that locally best approximates the tesseral layer. For a foreground voxel *x* ∈ *F*, let *H*_*x*_ be this best-fitting plane. Then, we define the 2D distance map *D*_2*D*_ as

$$\begin{array}{c} D_{2D}\left(x\right)=\{\begin{array}{cc} \min_{y\in \left(\Omega \setminus F\right)\cap H_{x}}∥x-y∥ & ,\;\;\text{if}\;x\in F\\ 0 & ,\;\;\text{if}\;x\notin F\;. \end{array} \end{array}$$

**Fig 5:**
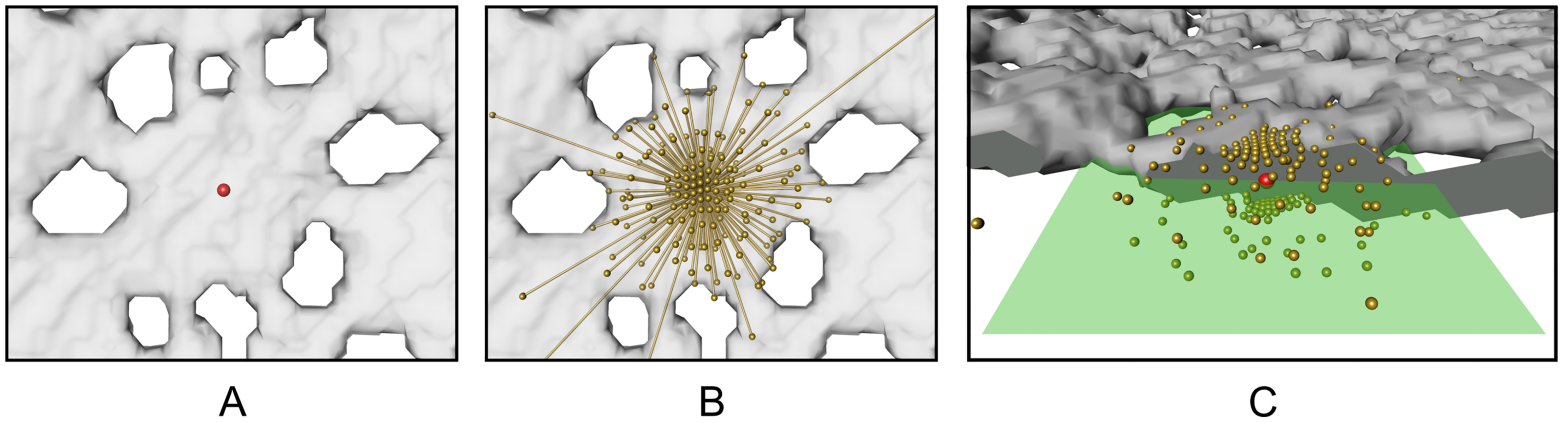
Plane approximation for 2D distance map computation. (A) The red point shows the position of the voxel *x* for which the plane approximation should be computed. The mineralized cartilage (foreground) is shown as transparent surface. (B) Rays starting from *x* and intersection points $P_{x}^{3D}$. (C) Rotated view showing the best fitting plane *H*_*x*_ (green) for the points shown in (B). The mineralized cartilage has been cut in order to visualize the orientation of the plane.

In order to compute the best-fitting plane *H*_*x*_ at voxel *x* ∈ *F*, we shoot *n* equally distributed rays in all directions in three-dimensional space (see Fig 5B). We compute the first intersection points of these rays with *Ω* \ *F*. Let $P_{x}^{3D}$ be the set of all these intersection points for rays starting in *x*. Then, we define ${\tilde{H}}_{x}$ as the plane with minimal squared Euclidean distances to all points in $P_{x}^{3D}$, that is

$$\begin{array}{c} {\tilde{H}}_{x}=\underset{H}{arg min}dist\left(H,P_{x}^{3D}\right)=\underset{H}{arg min}\sum_{q\in P_{x}^{3D}}dist{\left(H,q\right)}^{2}. \end{array}$$

*H*_*x*_ then is the plane parallel to ${\tilde{H}}_{x}$ with *x* ∈ *H*_*x*_ (see Fig 5C for *H*_*x*_). In order to compute *D*_2*D*_(*x*), we have to compute the intersection of *Ω* \ *F* and *H*_*x*_. Since this is computationally expensive we instead approximate *D*_2*D*_(*x*) by again using ray casting. In particular, we cast *m* equally distributed rays in *H*_*x*_ starting from *x*. For each of these rays, we measure the distance to the nearest background voxel. The shortest of these distances is then used for the 2D distance map at voxel *x*. Let $P_{x}^{2D}$ be the set of closest intersection points of the *m* rays with *Ω* \ *F*. Then, the approximate 2D distance map ${\tilde{D}}_{2D}$ is defined as

$$\begin{array}{c} {\tilde{D}}_{2D}\left(x\right)=\{\begin{array}{cc} \min_{y\in P_{x}^{2D}}∥x-y∥ & ,\;\;\text{if}\;x\in F\\ 0 & ,\;\;\text{if}\;x\notin F\;. \end{array} \end{array}$$

For the calculation of the intersections points with the background, the rays are traversed using the Bresenham algorithm [22].

#### Hierarchical watershed transform

The watershed transform [23] is named for the analogy of flooding a landscape, since gray values in an image can be thought of as topography. If a hole is drilled through the bottom of all basins of the landscape (i.e. the local minima of the gray-scale image), the rising water will fill the basins from the bottom. While the water continues to rise, water from different basins will meet at the edges, with the watersheds acting to keep basins separate. In this way, an image can be divided into distinct regions. The original watershed transform (first presented by Beucher and Lantuéjoul [23] with later improvements in speed, applicability and generality in [24–28])]), however, usually results in an oversegmentation, separating the image into too many small regions. To overcome this, a hierarchical watershed transform was proposed [29, 30], which starts from an oversegmentation and merges neighbored regions depending on different criteria, leading to a hierarchy of segmentations.

**Fig 6:**
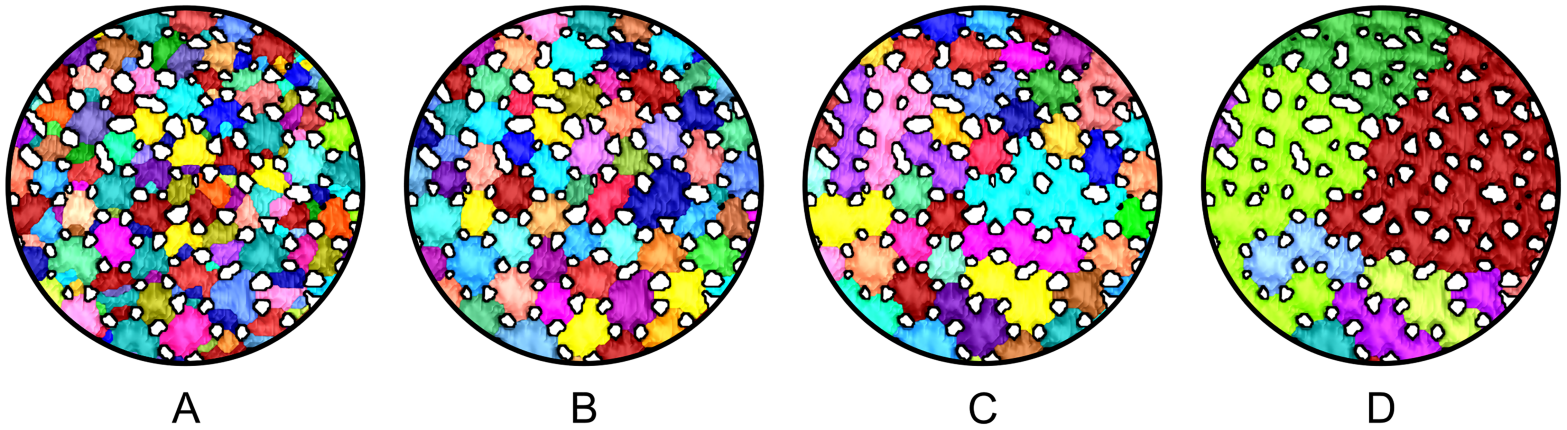
Illustration of the hierarchical watershed segmentation results. Different persistence thresholds were used: 0 (A); 10 (B); 30 (C); 50 (D). While (A) shows an oversegmentation, (B) represents an almost perfect segmentation. In (C) and (D), the chosen persistence thresholds were too large, resulting in undersegmented regions that comprise several tesserae.

In order to separate the tesserae from one another, we apply a hierarchical watershed transform on the inverted 2D distance map $-{\tilde{D}}_{2D}(x)$. In $-{\tilde{D}}_{2D}(x)$, the local minima are those foreground voxels with locally maximal distance to the background. In general, the local minimum will be at the centers or close to the centers of the tesserae. This is where the basins have their deepest points and from where the watershed transform starts. The watersheds itself are not explicitly created, instead neighbored basins are directly connected. Basins are flooded from each local minimum leading to an oversegmentation of *I*. The hierarchical watershed transform overcomes this oversegmentation problem by gradually merging neighbored regions. Several criteria can be used for merging. We employ a persistence-based approach by comparing the minima of the two neighbored regions with the value at the points where the two regions meet first during flooding. We call those points the saddle points. Merging is applied in ascending order of minimal value of the saddle points. Two regions are merged if the difference between the saddle point and either of the two minima is smaller than a user-defined persistence threshold *T*_*p*_. Additionally, regions are merged during the merging process if their number of voxels is lower than a predefined number of voxels threshold *T*_*v*_. Smaller persistence values lead to finer segmentations (see oversegmentation in Fig 6A) while higher persistence values lead to coarser segmentations (Fig 6B–6D). The result of this segmentation step will be called *hierarchical watershed segmentation* throughout this paper.

#### Proofreading

Even though the hierarchical watershed transform with a carefully chosen persistence value produces good results, as demonstrated later in the Evaluation section, some errors will always remain. We developed manual proofreading tools that allow easy and quick removal of the occurring errors.

**Fig 7:**
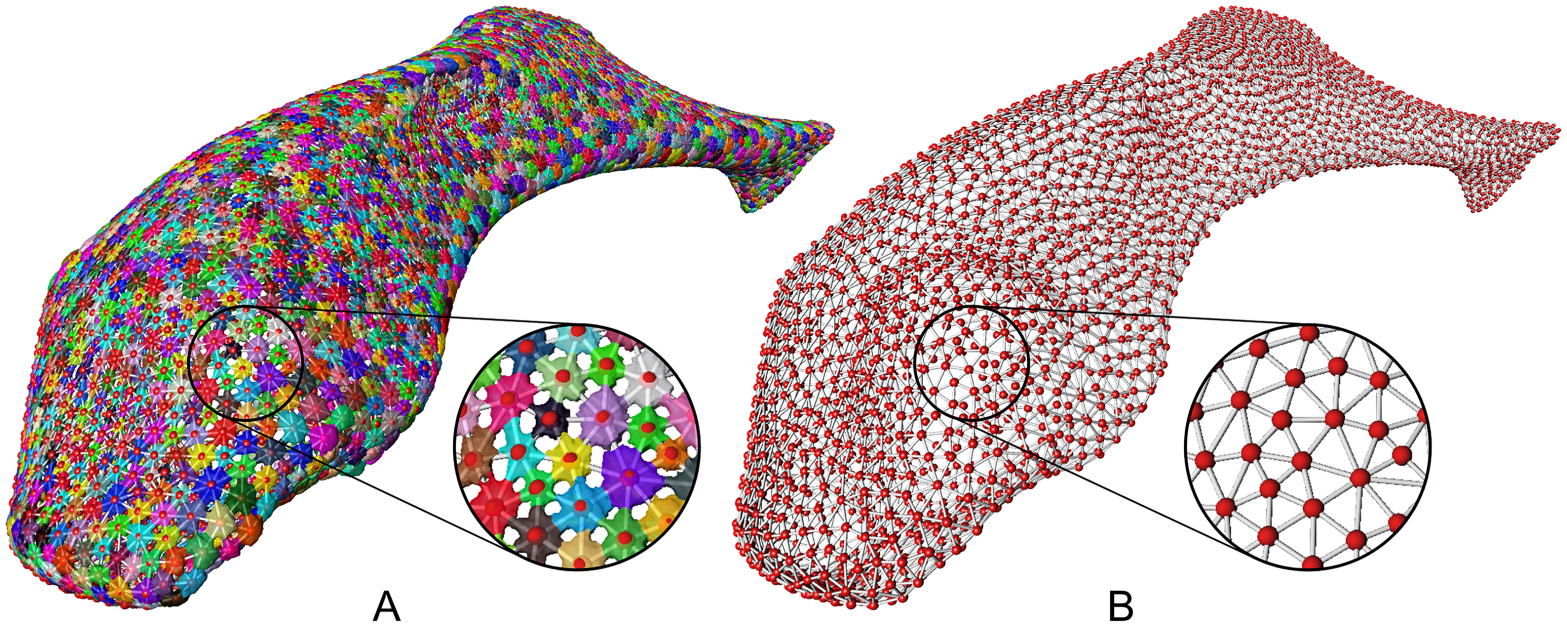
Graph representation of the segmentation. (A) Transparent surface and graph; (B) Only the graph. In the close-ups, the layer of vertices on the backside has been removed.

For this, we create a graph containing one vertex for each label (the red balls at the center of each tessera in Fig 7) and one edge (white linear elements in Fig 7) connecting two vertices if their corresponding label regions are in contact. This graph is used for three main purposes: First, it enables easy identification of segmentation errors because the tesserae network is usually highly regular. Any irregularity in the network might indicate an error. Second, it allows comfortable user interaction. The user can select regions by selecting the corresponding graph vertices. Third, it is a useful representation to depict statistical data—such as a tessera’s volume, number of neighbors, or surrounding surface curvature—which can be directly mapped onto the vertices or edges. This functionality is vital for multi-variate, quantitative analysis of the biological data that will be presented in a further publication.

**Fig 8:**
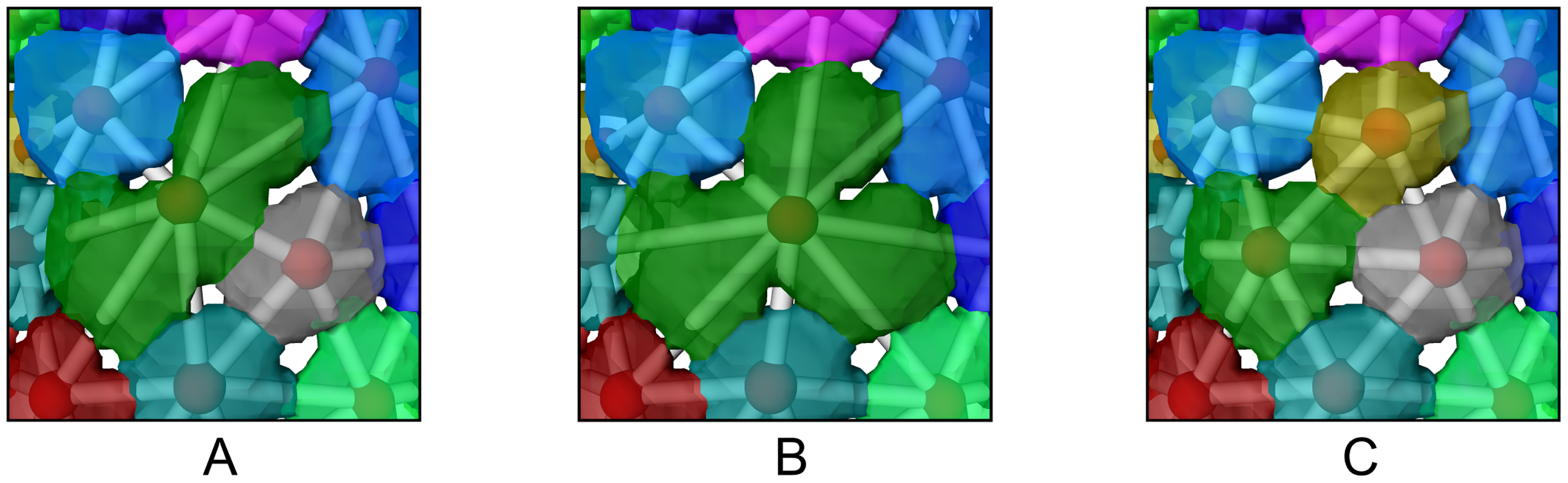
Example for manual error correction using a combination of merge and split operations. Additionally, the graph representation is shown with vertices inside a transparent surface. (A) There are three tesserae without pores between them, this leads to a bad watershed segmentation result; (B) Merge the three labels into one large label; (C) Use spectral-clustering-based split to resolve the error. Note how the corrected graph is more regular regarding edge lengths and vertex positions compared to (A) and (B).

The error correction works on an inter-region level of detail, that means, the user does not have to deal with individual voxels but can select label regions via the graph representation and start error correction methods directly on the regions. There are mainly three kinds of errors: First, one tessera is split incorrectly into multiple regions (oversegmentation). Here the user selects the regions belonging to a single tessera and merges them into one. Second, two or more tesserae are represented by one label (undersegmentation). Here, the user can select this label region and apply a split operation. Currently two split operations are available: (1) watershed-based; (2) spectral clustering-based (see [31] for a good introduction). In both cases, only voxels belonging to the selected region are taken into account. Third, tesserae share a badly shaped boundary. Here, a merge operation followed by a split can be used to correct the problem. Fig 8 shows how to efficiently resolve a complex problem created by a missing pore space between tesserae by combining a merge step with a split step. Such methods are necessary because we are working with complex biological data and not all parts of the input image follow our idealized flat tesserae shape with gaps between them.

For error detection we use two further possibilities next to the already mentioned graph irregularities. First, we can simply look for errors by visually comparing the segmentation with the image *I*. Second, we use statistical information based on the segmentation. For each label we compute the volume, number of neighbors, distance of a vertex to its nearest vertex, width, height and curvature, where the curvature is computed using the mesh structure of the graph. Labels with unusual values (i.e. labels with very large or very small volume) contain potential segmentation errors. Since tesserae can have large differences regarding their size and shape, we do not apply correction algorithms automatically but rely on automatically-guided manual corrections supported by the mentioned split and merge operations.

The segmentation resulting after this postprocessing step will be called *final pipeline segmentation* throughout this paper.

### Computational costs and parameters

All segmentation steps are performed in the visualization software Amira [32] with either off-the-shelf modules or modules specifically implemented for this task.

#### Remarks on implementation and computational costs

Let |*I*| be the overall number of voxels in image *I*, |*S*| the number of voxels belonging to the user-defined region *S*, and |*F*| the number of foreground voxels. Then, the computational costs for each step of our pipeline can be given as follows.

1. *Anisotropic diffusion*: In each timestep, we have a complexity of *O*(|*I*|) (big *O* notation for time complexity, see, for example, [33]). We use CUDA to parallelize the computations in each timestep.
2. *Average computation for local thresholding*: We only need to consider voxels belonging to the user-defined area *S*. For those voxels, we compute the neighborhood average values for the first xy-slice. Then we iterate over increasing z-values in parallel on the CPU, i.e. with one thread for one pair of *x* and *y* coordinates. Here, we reuse the result of the computation at voxel (*x*, *y*, *z* − 1), if available, for the computation at (*x*, *y*, *z*). In this case, the computational cost for a voxel in *S* reduces from *O*(*l*^3^) to *O*(*l*^2^), where *l* is the cube length of *B*(*x*, *l*).
3. *2D distance transform*: We iterate over all foreground voxels, which is done in parallel on the CPU. For each foreground voxel, we shoot *m* 3D and *n* 2D rays in the foreground. Since the rays terminate early, in practice the costs for each voxel are *O*(*m*) and *O*(*n*), respectively. Hence, the overall complexity is *O*((*m* + *n*) ⋅ |*F*|).
4. *Hierarchical watershed transform*: In the initialization, which is only performed once, we need to sort all foreground voxels. This step has a complexity of *O*(|*F*| ⋅ log|*F*|). It is the most costly step in computing the Watershed transform. Afterwards, for watershed segmentations on the same data but with different persistence values, we only have to iterate over the label regions created for a persistence value of 0.
5. *Graph computation for manual proofreading*: We iterate over all foreground voxels, which again is done in parallel on the CPU. This has a complexity of *O*(|*F*|).

#### Parameters

In this section, we describe how suitable parameters can be found for the respective steps of our pipeline.

**Fig 9:**
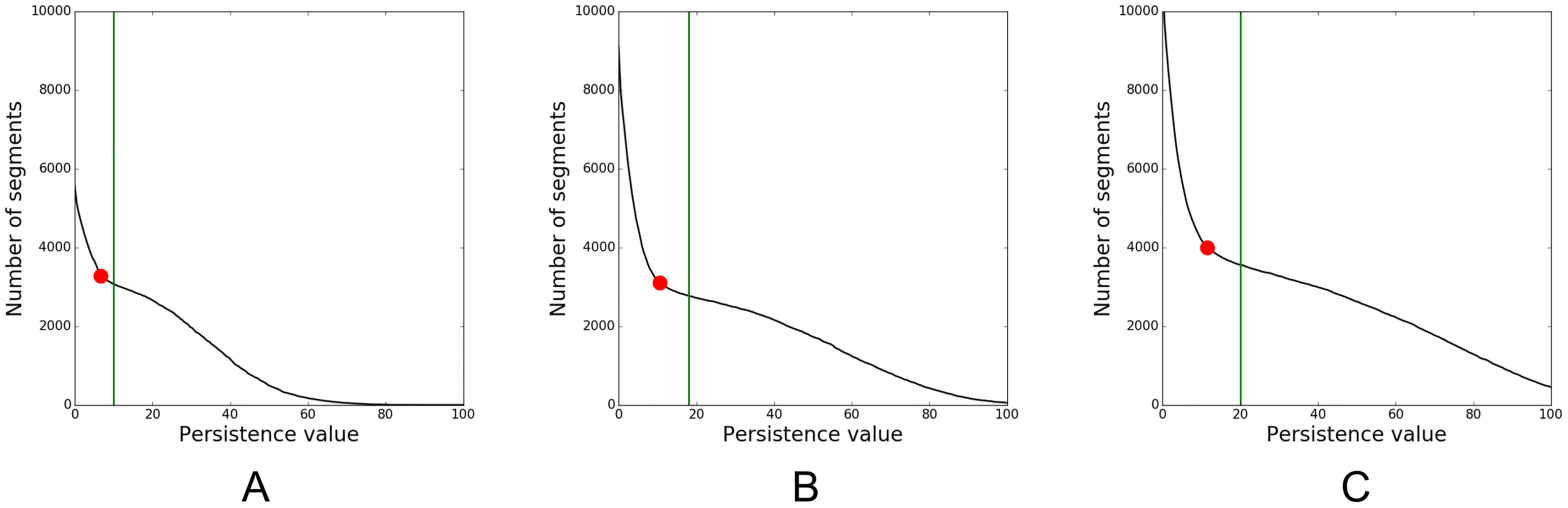
Number of labels created by hierarchical watershed segmentations plotted against persistence values. Computations are done for three hyomandibula datasets with increasing hyomandibula size for persistence values from 0 to 100. The green lines indicate the persistence value we chose for the hierarchical watershed segmentations (A: 10, B: 18, C: 20). Note how the values increase with hyomandibula size.

1. Anisotropic diffusion
  - Diffusion stop threshold *T*_*s*_: Calculate the range of gray value differences between tessera voxels and adjacent background voxels, and between tesserae and intra-tesseral holes. Choose a value smaller than the former values and larger than the latter values.
  - Time *t*: Choose a small timestep and check whether intra-tesseral holes have been removed. Otherwise increase the value and repeat the procedure.
2. Local thresholding
  - Box length *l*: Set *l* to approximately two to three times the average tessera thickness. In this way the box *B*(*x*, *l*) around a voxel *x* in the center of a tessera will contain a sufficient number of background voxels.
  - Strip *S* around tesserae: Generate a strip *S* containing foreground voxels and near background voxels such that boxes *B*(*x*, *l*) around *x* ∈ *S* contain foreground and background voxels. That means *x* ∈ *S* cannot be further away than *l*/2 from the nearest tessera voxels. To compute *S*, perform a rough foreground segmentation by simple thresholding. The resulting label region should contain all foreground voxels. Now perform region growing to reach the desired size of *S*.
  - Threshold *T*_*n*_: Test multiple values near 1 and visually evaluate the foreground segmentation quality. For the results presented in this paper, we used *T* = 1.
3. 2D distance transform
  - Number *n* of 3D rays to compute the plane *H*_*x*_: Set *n* large enough to deal with outliers; for the results presented in this paper, we used *n* = 1214, but a smaller number should already suffice. Enlarge *n* if the resulting plane is inaccurate.
  - Number *m* of 2D rays shot in the plane *H*_*x*_: Set *m* large enough to hit pore space; for the results presented in this paper, we used *m* = 361, but a smaller number should suffice.
4. Hierarchical watershed transform
  - Number of voxels threshold value *T*_*v*_: Estimate the number of voxels in the smallest tesserae and take a value for *T*_*v*_ smaller than that.
  - Persistence value *T*_*p*_: Plot the number of labels versus the persistence value (Fig 9). As can be seen in such plots, the number of labels drops off rapidly initially at low persistence values, before leveling off at a certain inflection point (shown as red dot). The persistence thresholds after this first sharp turn are good candidates. The final persistence value can be found by visually inspecting the segmentation results and comparing it with an isosurface or volume rendering of *I*.

## Evaluation

For the evaluation, we applied our segmentation pipeline to μCT scans of the right hyomandibulae of three ages of stingray that were already described in the ‘Input Data’ section of ‘Materials and Methods’. These hyomandibula datasets are called I, II and III throughout this section, increasing in physical size from I to III. They each consist of thousands of tesserae and have a voxel size of 9.78 μm after resampling. For further information see Table 1.

**Table 1:** Hyomandibula dataset information.

Dataset	Disc width in cm	Size in voxels	Image resolution in μm
I	11.0	558 x 495 x 1385	9.78
II	14.4	785 x 535 x 1873	9.78
III	19.0	875 x 893 x 2477	9.78

Disc width is a common measure for stingray size and age.

Calculations were carried out on a desktop PC with two Intel Xeon E5-2650 processors (each 8 cores with 2.6 GHz), 128 GB RAM, and a GeForce GTX 780 Ti. Dataset I required approximately the following computation times (pure running times of the algorithms without user interaction and parameter finding): *Anisotropic diffusion*: 17; *Average computation for local thresholding*: 26; *2D distance transform*: 12; *Initialization of hierarchical watershed transform*: 20; *Graph computation for manual proofreading*: 3. Including parameter finding, the creation of the hierarchical watershed segmentation required approximately one hour of work. The time for the manual corrections depends on the required segmentation quality. We were able to create high-quality segmentations in approximately two hours starting from a well-chosen hierarchical watershed segmentation.

For the datasets I, II, and III, we computed the hierarchical watershed segmentations for persistence values from 0 up to 80 in single steps using a minimum number of voxels threshold *T*_*v*_ of 50 for datasets I and II, and a value of 100 for dataset III. We used a persistence value of 10 for I, of 18 for II and of 20 for III (Fig 9) to create the initial hierarchical watershed segmentations that we improved in our last pipeline step to create the final pipeline segmentations. Additionally, we computed hierarchical watershed segmentations on the standard three-dimensional (3D) distance map *D*_3*D*_, defined as

$$\begin{array}{c} D_{3D}\left(x\right)=\{\begin{array}{cc} \min_{y\in \Omega \setminus F}∥x-y∥ & ,\;\;\text{if}\;x\in F\\ 0 & ,\;\;\text{if}\;x\notin F\;, \end{array} \end{array}$$

to allow a quantitative comparison with the segmentation results obtained using the 2D distance map.

### Qualitative evaluation

Visual comparison of the segmentation result with an isosurface, a volume rendering or slices of the μCT image allowed qualitative evaluation. This was mainly used during the postprocessing part of the pipeline for fast error correction.

### Quantitative evaluation

Quantitative evaluation of the segmentation pipeline was performed in two ways: (1) by comparing hierarchical watershed segmentations for varying persistence values and the final manually improved pipeline segmentation with manually placed landmarks; (2) by comparing the final pipeline segmentation with regions manually segmented by several users.

#### Landmark-based evaluation

**Fig 10:**
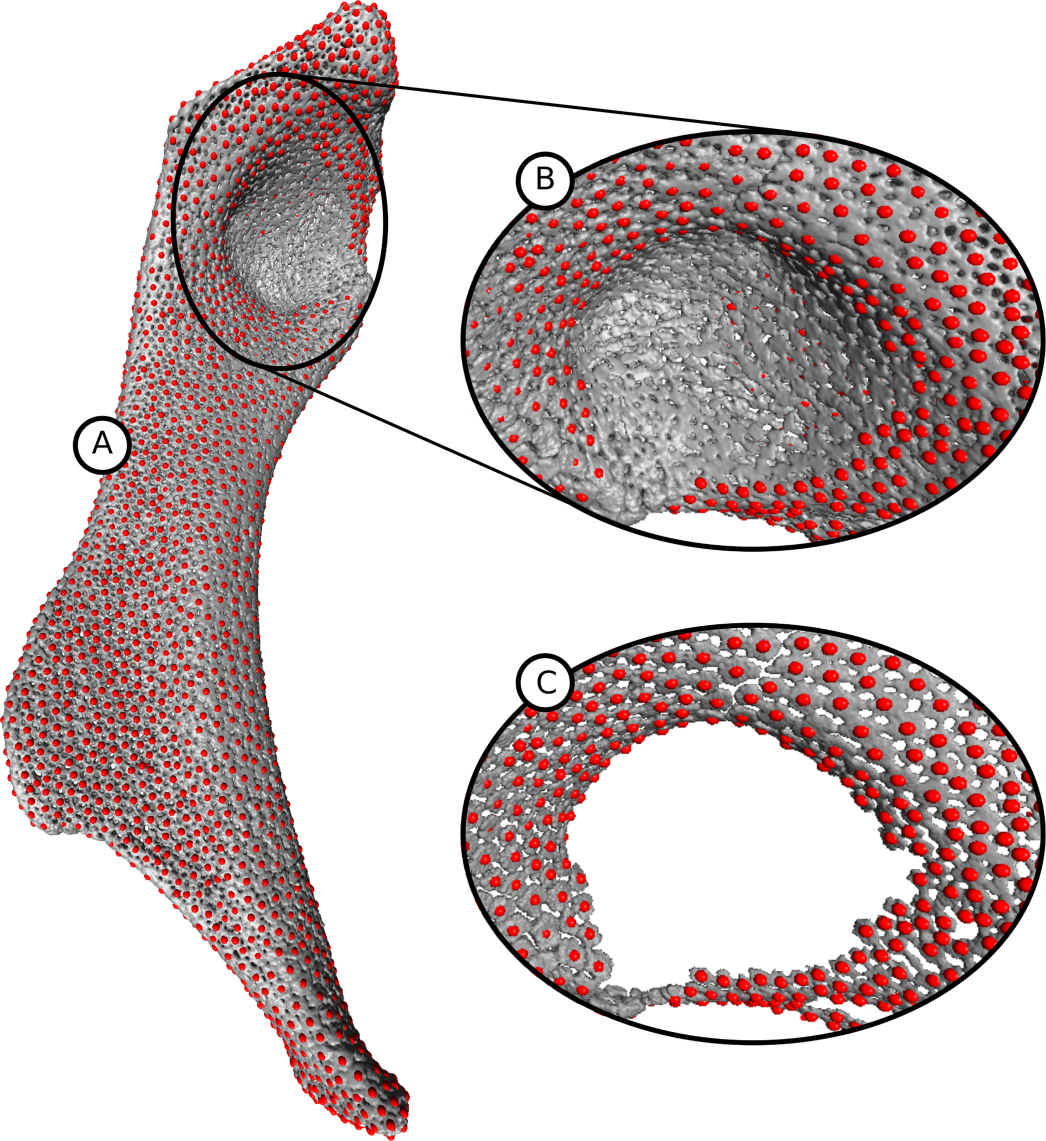
Manual landmarks for evaluation. (A) Isosurface of dataset I with one manually placed landmark per tessera but without landmarks on low mineralized areas. (B) Rotated close-up of the region where the tendon is connected to the hyomandibula. Here, no landmarks were created because it is very difficult or impossible to distinguish the tesserae in this region. (C) Same close-up as in (B) but without the low mineralized regions. The backside of the hyomandibula was removed for this image.

For the first part of the evaluation, landmarks were placed manually near the centers of all tesserae on isosurface renderings of the three datasets I, II, and III; for several thousand tesserae per hyomandibula, this required approximately five hours per dataset. Regions with little or no mineralization (e.g. the area where a tendon inserts into a large anterior fossa in the hyomandibula) were excluded from the analysis, because even for domain experts (i.e. researchers very familiar with the tissue), it is not possible to identify individual tesserae, see Fig 10. The number of created tesserae and the number of manually placed landmarks are stated in Table 2.

**Table 2:** Number of tesserae in 2D distance map-based final pipeline segmentations.

Dataset	Final pipeline segmentation	Final pipeline segmentation	Number of manual landmarks
		excluding low mineralized areas	
I	3081	2769	2746
II	2759	2385	2364
III	3488	3048	3032

In order to evaluate a segmentation, we compute precision and recall values with the help of the landmarks. A label region that is hit by at least one manually placed landmark is a true positive, a region with *n* hits leads to *n* − 1 false negatives (an *n*-cluster that requires a split operation) and a region with zero hits is a false positive (oversegmentation that requires a merge operation). Let *t*_*p*_, *f*_*n*_, and *f*_*p*_ be the number of true positives, false negatives and false positives, respectively, then precision and recall can be computed as follows:

$$\begin{array}{c} \text{precision}=\frac{t_{p}}{t_{p}+f_{p}}\;\text{and}\;\text{recall}=\frac{t_{p}}{t_{p}+f_{n}}\;. \end{array}$$

Since manual creation of thousands of landmarks is error-prone, we double-checked the correctness of all landmarks belonging to false positive and false negative regions.

**Fig 11:**
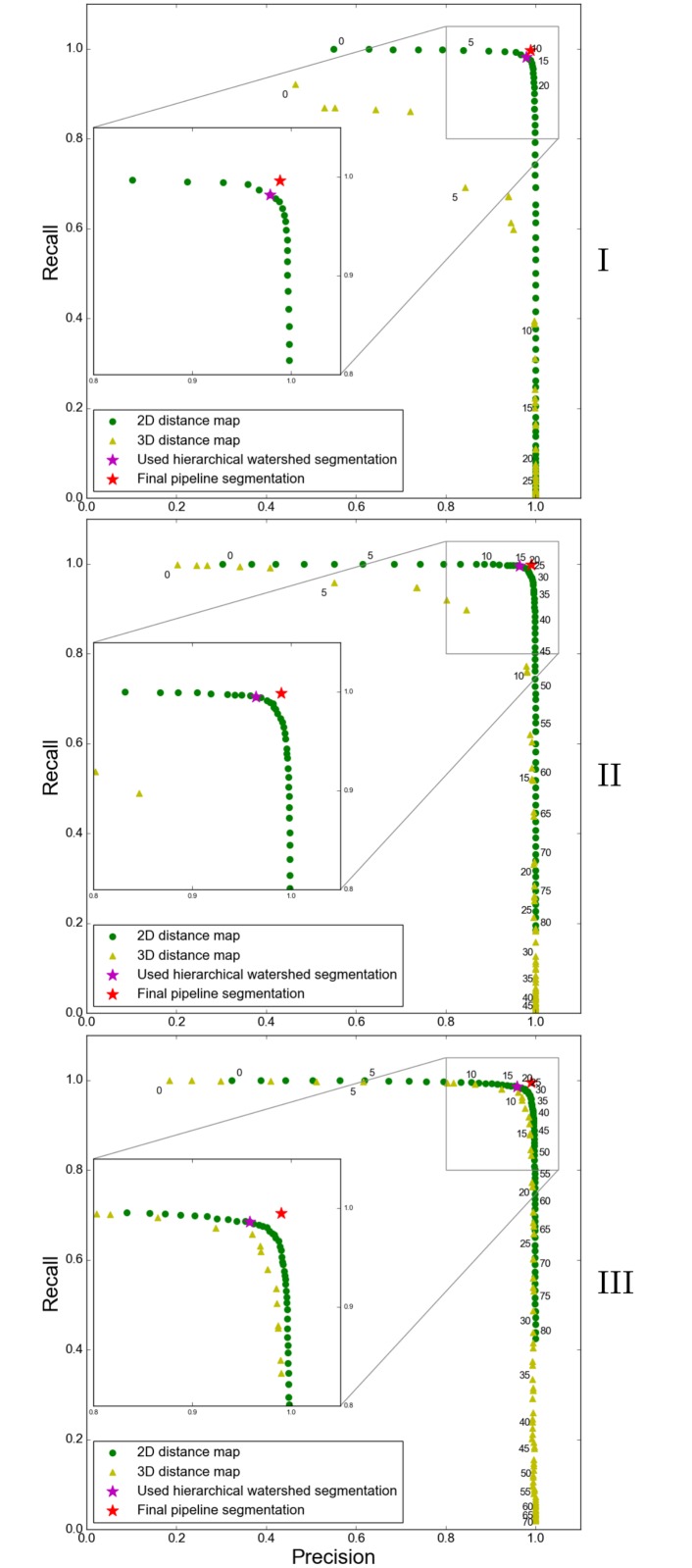
Precision-recall plots for datasets I, II and III. Persistence values range from 0 to 80 in single steps. Each fifth persistence value is written at the top right position next to the respective green dot in case of the 2D distance map and at the bottom left position of a yellow triangle in case of the 3D distance map. The final pipeline segmentation is highlighted with a red star (I: precision 0.9888, recall 0.9964; II: 0.9899, 0.9987; III: 0.9898, 0.995), the watershed segmentation used to generate this final pipeline segmentation is highlighted with a magenta star (I: precision 0.9786, recall 0.9822; II: 0.9643, 0.9949; III: 0.9583, 0.9864).

Fig 11 shows the precision-recall plots for the 2D and 3D distance map. The hierarchical watershed segmentation used to generate the final pipeline segmentation is shown by a magenta star, whereas the final pipeline segmentation is indicated by a red star. Note that for the chosen hierarchical watershed segmentation, a slightly better recall value is preferable compared to the precision value because errors leading to a worse precision value can usually be corrected by merge operations, which are easier to perform than split operations. Fig 11 shows that the 3D distance map results in very bad segmentations for datasets I and II (for chosen persistence values no precision/recall pair with both values higher than 0.8 in I and only two such pairs in II where the precision values are smaller than 0.85). In III the 3D distance map leads to better values compared to I and II (14 precision/recall pairs with both values higher than 0.8) but the results are still inferior to the 2D approach. On the other hand, the 2D distance map leads to multiple precision/recall pairs with values higher than 0.95 for all three regions.

**Fig 12:**
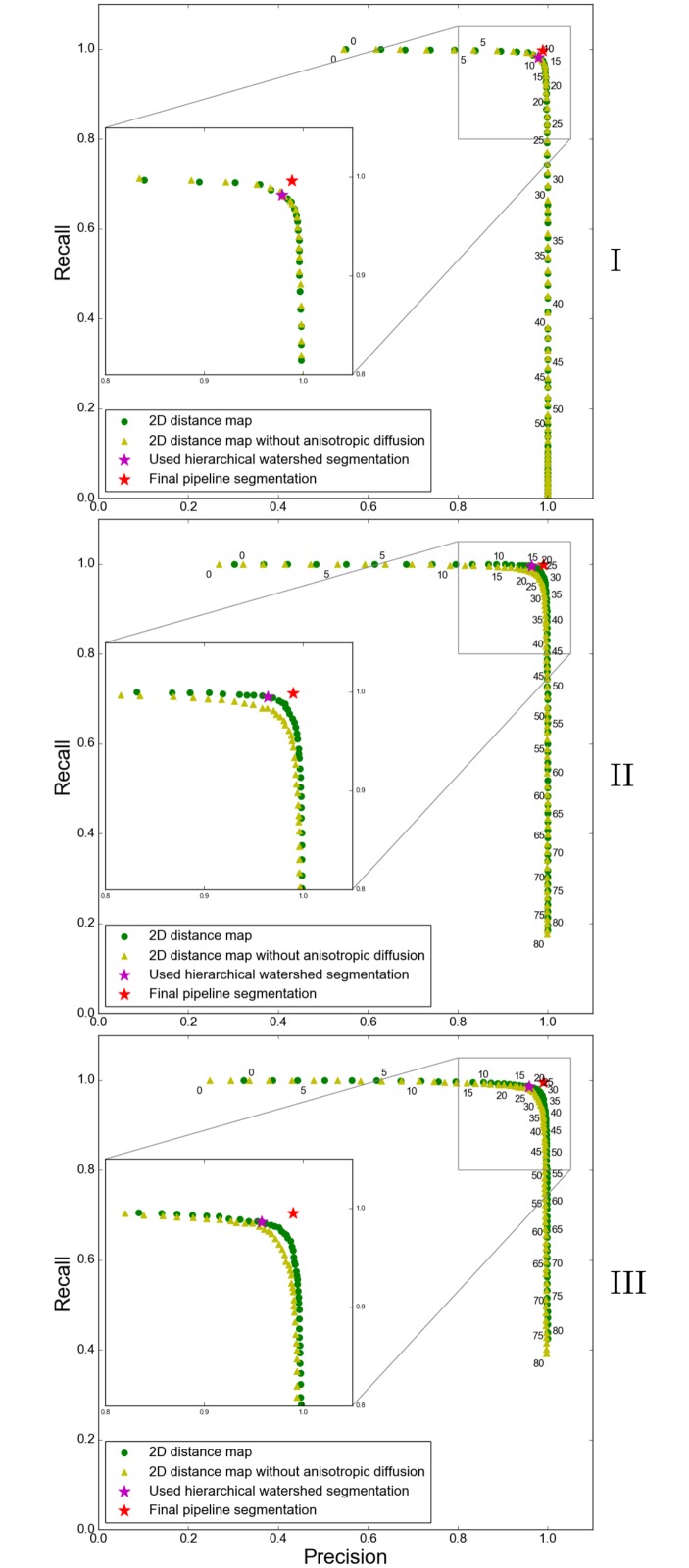
Precision-recall plots for datasets I, II, and III with and without preprocessing (anisotropic diffusion). Each fifth persistence value is written at the top right position next to the respective green dot in case of a 2D distance map with preprocessing and at the bottom left position of a yellow triangle in case of a 2D distance map without preprocessing. The final pipeline segmentation is highlighted with a red star, the watershed segmentation used to generate this final pipeline segmentation is highlighted with a magenta star.

Furthermore, we want to highlight the importance of anisotropic diffusion as a preprocessing step. Fig 12 compares the precision-recall values for the 2D distance map with and without anisotropic diffusion. ‘Without anisotropic diffusion’ means that we skipped the preprocessing step and started the pipeline with the local thresholding working on image *I* instead of *I*_*s*_. While there is no improvement in dataset I, the results for datasets II and III considerably improve by applying anisotropic diffusion.

#### Region-based comparison with manual segmentations

**Fig 13:**
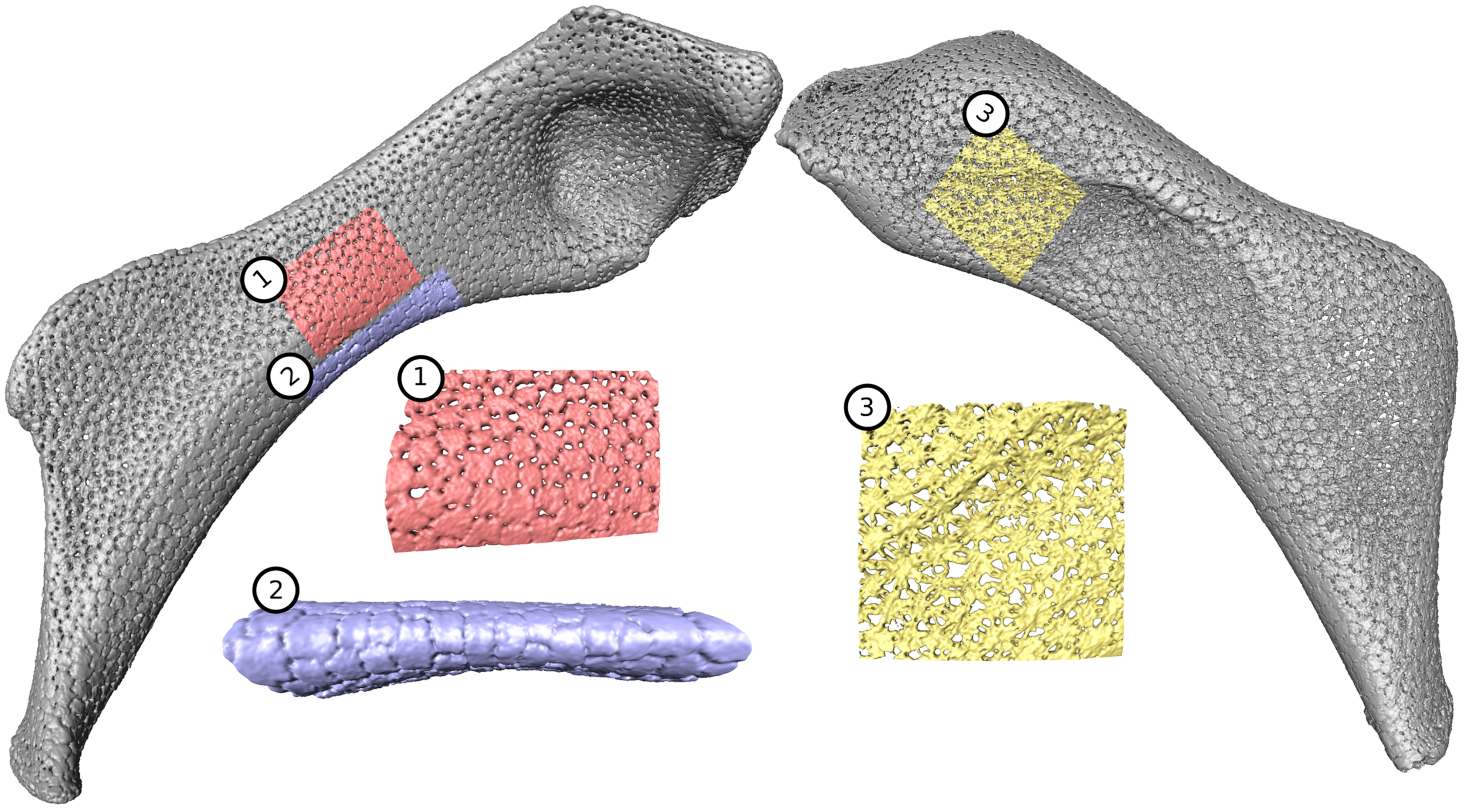
Selected regions for manual segmentations. (1) Flat regularly-shaped tesserae with bounding box size of 120 x 84 x 178 taken from dataset I containing 99 tesserae; (2) Edge region with bounding box size 94 x 195 x 254 taken from dataset I containing 83 tesserae; (3) Flat region with thin, irregularly-shaped tesserae, perforated by large pores and intra-tesseral holes with bounding box size 215 x 126 x 228 taken from dataset II containing 85 tesserae.

Landmark-based evaluation does not detect errors concerning the shape of tesserae. Therefore, in the second part of the evaluation, we compared one final pipeline segmentation with manual segmentations independently created by four persons whereby two of them were domain experts. Correct ground truth segmentations are not available for our problem. We used the RAND index [34] and the variation of information (VI) [35] measure for comparison. Because manual segmentation of a whole dataset would have been too time-consuming, we chose three regions which were representative of different types of tesserae seen in our datasets (see Fig 13). Each of those regions could be manually segmented in approximately one to three hours, which would lead to an extrapolated manual segmentation time between 33 and 100 hours for one complete hyomandibula (assuming the region contains 90 tesserae and the hyomandibula consists of 3000 tesserae). The first region is flat with regularly-shaped tesserae (Fig 13A), the second region contains an edge region (region with high curvature) of the hyomandibula (Fig 13B), and the third region is also flat but thinner and consists of less mineralized, irregularly-shaped tesserae (Fig 13C).

In this evaluation step we were not interested in differences between foreground and background. Thus only voxels belonging to the foreground in all segmentations were taken into account. This is a safe assumption because the manual segmentations used a threshold to separate foreground from background and if necessary it is easy to enlarge a given segmentation to include more background voxels near the label boundaries by using region growing.

**Fig 14:**
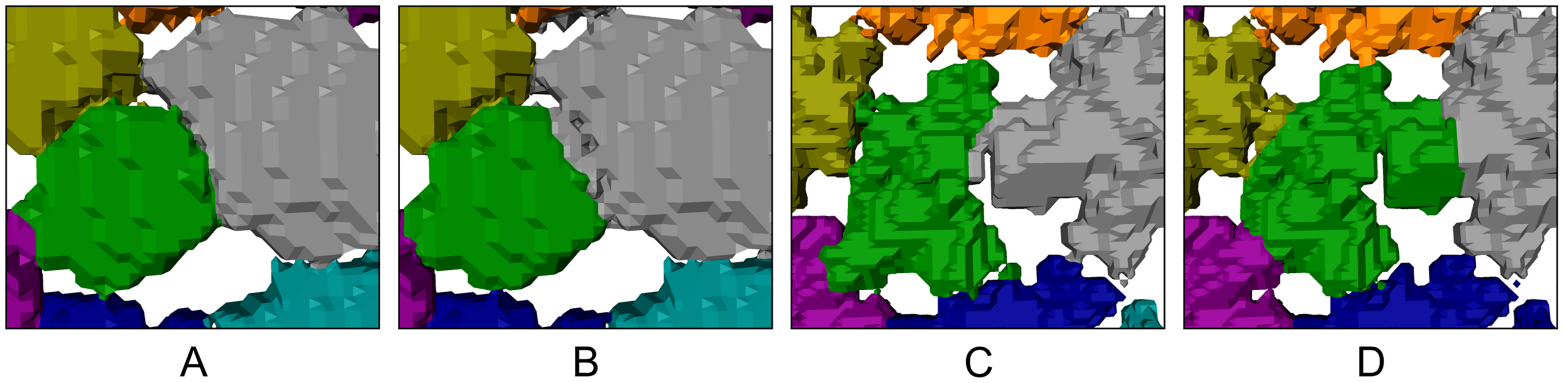
Close-up showing the worst VI label pair for region 1 (A/B) and region 3 (C/D). (A) Final pipeline segmentation of region 1; (B) Manual segmentation 1 of region 1; (C) Final pipeline segmentation of region 3; (D) Manual segmentation 1 of region 3.

The RAND and VI values are shown in Tables 3, 4 and 5. We compared the final manually corrected pipeline segmentation with all manual segmentations and also all manual segmentations with each other. Region 1 fulfills our dataset conditions, that means it is a flat area where the thinnest part of the inter-tesseral connection determines the correct border. Here, the differences between the final pipeline segmentation and the manual segmentations are insignificantly larger or in some cases even smaller (i.e. final pipeline segmentation with manual segmentation 2 compared to manual segmentation 1 with manual segmentation 3) compared to the differences among the manual segmentations. Because region 2 is an edge region (not flat, problematic for the 2D distance map) and region 3 consists of irregularly-shaped tesserae (thinnest parts of inter-tesseral connections are not always determining the correct border), the differences between the final pipeline segmentation and the manual segmentations are larger compared to region 1, but the values still indicate a good segmentation result. As an example, in Fig 14 we show the two labels that contribute the largest error (the two labels that add the largest value to the sum over all label pairs) into the VI computation in region 1 and region 3 for the comparison between the final pipeline segmentation and manual segmentation 1. For region 1, the algorithmic segmentation is even better, the VI value rises because of errors in the manual segmentation (see Fig 14A and 14B). Fig 14C and 14D highlights the irregularity of region 3.

**Table 3:** VI / RAND values for region 1 consisting of flat regularly-shaped tesserae.

	Final	Manual 1	Manual 2	Manual 3
Final	-	-	-	-
Manual 1	0.230 / 99.8764	-	-	-
Manual 2	0.209 / 99.8826	0.205 / 99.8826	-	-
Manual 3	0.207 / 99.8844	0.223 / 99.8628	0.176 / 99.9037	-
Manual 4	0.206 / 99.8696	0.228 / 99.8484	0.174 / 99.8809	0.183 / 99.8758

**Table 4:** VI / RAND values for region 2 (edge region).

	Final	Manual 1	Manual 2	Manual 3
Final	-	-	-	-
Manual 1	0.252 / 99.7975	-	-	-
Manual 2	0.202 / 99.8405	0.197 / 99.8526	-	-
Manual 3	0.248 / 99.7978	0.235 / 99.8122	0.180 / 99.8645	-
Manual 4	0.221 / 99.8158	0.219 / 99.8235	0.166 / 99.8679	0.203 / 99.8460

**Table 5:** VI / RAND values for region 3 consisting of flat tesserae with thin, irregularly-shaped tesserae, perforated by large pores and intra-tesseral holes.

	Final	Manual 1	Manual 2	Manual 3
Final	-	-	-	-
Manual 1	0.458 / 99.7084	-	-	-
Manual 2	0.422 / 99.7463	0.321 / 99.7995	-	-
Manual 3	0.401 / 99.7610	0.295 / 99.8152	0.266 / 99.8509	-
Manual 4	0.398 / 99.7542	0.288 / 99.8294	0.272 / 99.8357	0.192 / 99.8888

## Discussion

We have presented a pipeline for the semi-automatic segmentation of complex 3D structures tiled by repeating elements, demonstrating the pipeline’s efficiency and utility in the segmentation of the endoskeletal tesserae of sharks and rays and allowing the first quantification of the number of tesserae covering entire skeletal elements. The pipeline allows the rapid and tailorable computation of a variety of variables over multiple large datasets (e.g. entire skeletal elements), producing results nearly as accurate as manual approaches, but with significant improvements in speed. Our pipeline builds off of the central idea of using a watershed segmentation on a distance transform, and is highly modular, consisting of interchangeable steps that were fine-tuned to deal with tessera-specific problems. Because tessellated structures are common in biology, the following paragraphs will outline possibilities to adapt pipeline steps for other tessellated data, while also highlighting reasons behind specific tessera-related choices used in the current analysis.

**Fig 15:**
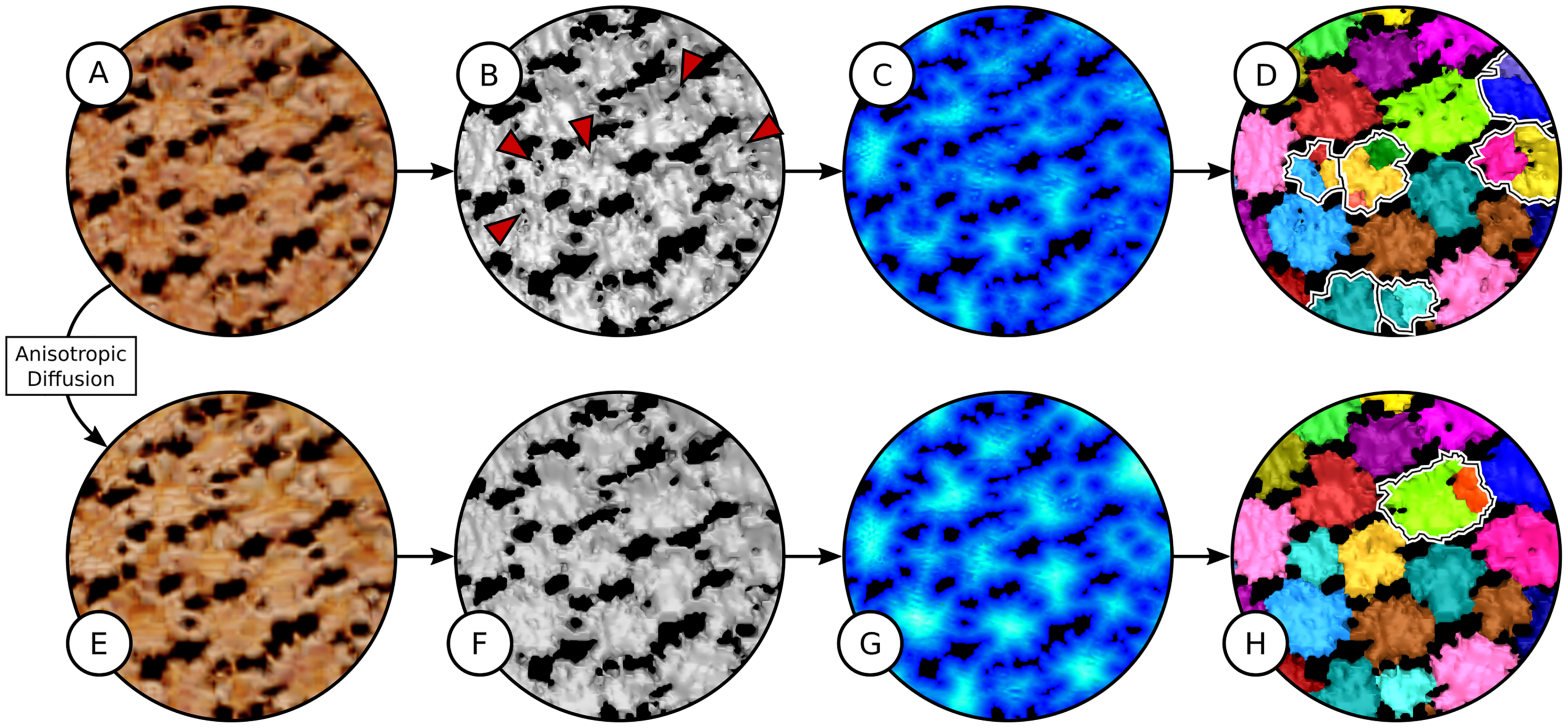
Comparison of results without (top row) and with (bottom row) anisotropic diffusion. (A,E) Volume rendering of original scalar field (A) and scalar field after anisotropic diffusion (E). (B,F) Surfaces of binary segmentations using the same parameters for local thresholding. The red arrow indicates intra-tesseral holes that appear in the binary segmentation if no anisotropic diffusion is applied. (C,G) Maximum intensity projections of 2D distance maps generated from binary segmentations. (D,H) Surfaces of segmentation results using comparable persistence parameters. Wrong segmentations are highlighted with outlines. In rare cases, the application of anisotropic diffusion might break tesserae (H), but these broken tesserae are usually easy to fix with a simple manual merge operation.

Scan data from both plant and animal tissues regularly exhibit porosity due to embedded cells and vasculature. **Anisotropic diffusion** as a preprocessing step is useful for porous data where problematic gray value differences inside of objects can be removed while keeping sharp outer edges. More sophisticated filters like non-local means [36] are able to smooth the data while maintaining important fine structures. The removal of problematic gray value differences is particularly important, since distance transforms are vulnerable to false gaps. This can exemplarily be seen for tesserae in Fig 15. Note that even though the effect of anisotropic diffusion can hardly be seen in the volume rendering of the gray-scale image (Fig 15A and 15E), it has an immense effect on the binary segmentation highlighted by the red arrows in Fig 15B.

A high-quality **foreground segmentation** is crucial for a successful distance map computation. For many datasets, global thresholding is sufficient. However, for datasets with varying intensity values for similar materials, a local thresholding approach is required. We employed our own special-purpose algorithm; for an overview about common local thresholding methods see [37].

**Fig 16:**
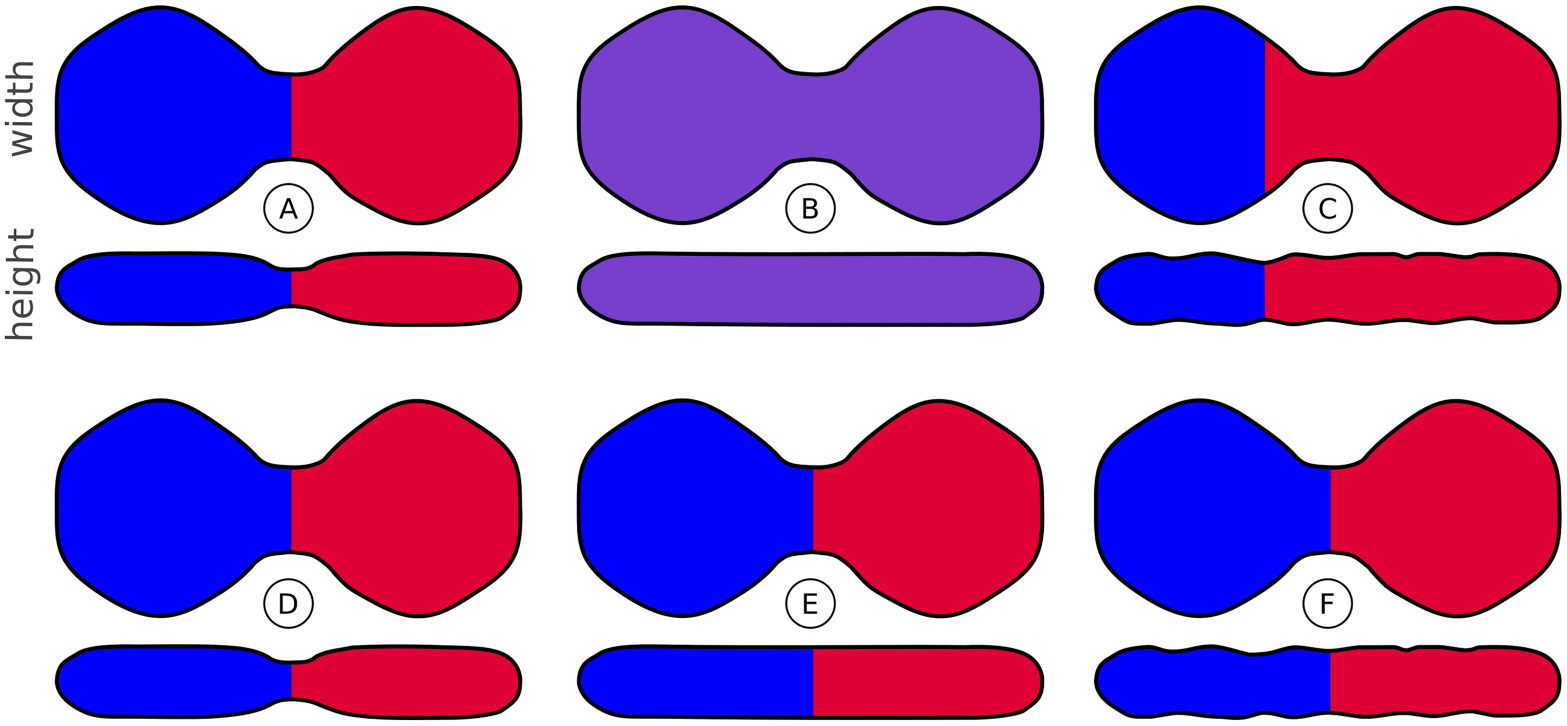
Illustration of segmentation problems: 3D distance map (top row) compared to 2D distance map (bottom row). (A-C) Segmentation based on 3D distance map for different cross sections. Only for (A) it is possible to generate the desirable segmentation. (B) The 3D distance map has only one maximum, hence no separation is possible. (C) The 3D distance map has several maxima but the separation appears at the wrong place, that is, where the object is thinnest. (D-E) Segmentation based on 2D distance map. Segmentation is successful for all three cross sections.

The choice of **distance map** depends strongly on the shape and morphology of the objects to be separated. The most common one is the standard 3D version, for which we have shown in Fig 11 that the segmentation results are inferior to our proposed 2D distance map in the case of tesserae segmentation. This is due to the following reasons. First of all, the 3D distance map bears some fundamental problems if the size of the interconnections is larger than one of the dimensions of the objects themselves. In this case, the 3D distance map measures the extension of the object regarding this dimension, but not the distance to the nearest pore. Two problems are illustrated in Fig 16B and 16C. Fig 16B shows a case in which no separation is possible because the height of the tesserae is smaller than the width of the interconnections and the tesserae cannot be separated by considering the height alone. In Fig 16C, the tesserae are separated at the wrong position, because the smallest height is not at the interconnection of the tesserae. In contrast, the 2D distance map results in a correct separation in all cases.

**Fig 17:**
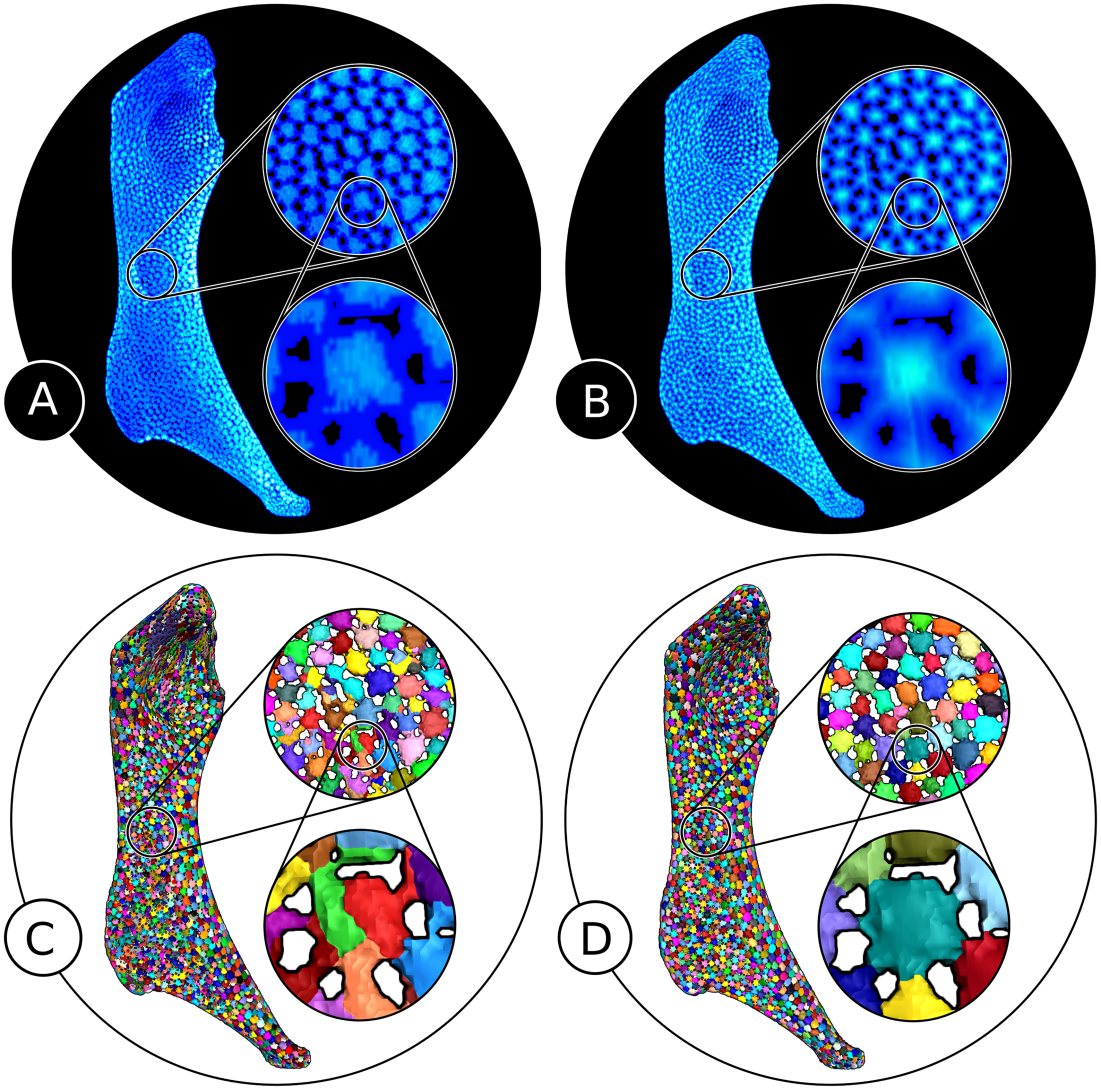
Comparison of 3D (A) and 2D (B) distance maps and resulting hierarchical watershed segmentations (C, D). (A) and (B) show maximum intensity projections of the 3D and 2D distance maps, respectively. Two zoom-in levels are provided to better illustrate the differences. (A) The 3D distance map tends to create large plateaus (i.e. areas with equal values), in particular in the regions of inter-tesseral connections, where projections of mineralized tissue are narrow in two dimensions. (C) These plateaus can span inter-tesseral joints, leading to inaccurate separation of individual tesserae. (B) By comparison with the 3D distance map, the 2D distance map has much more gradual contours and does not show any plateaus. Instead, the distance values decrease toward the inter-tesseral connections, allowing tesserae to be accurately separated well from one another, even when joint spaces are not evident (D).

Both cases illustrated in Fig 16B and 16C occur in actual datasets. Fig 17 examines these effects, showing segmentation results based on the 3D distance map in comparison to the 2D distance map. Note how the segmentation completely fails for the 3D case in Fig 17C, with errors that cannot be corrected by simple region merging. This is mainly due to the fact that the 3D distance map measures the height of the tesserae instead of the distance to the pores.

In the edge regions of the hyomandibulae that have high values for the primary principal curvature (e.g. region 2 in Fig 13), the tesserae geometries do not meet the shape assumptions underlying the use of the 2D distance map. Therefore, in those regions the number of oversegmentations compared to the number of correct segmented labels is higher than in the remaining regions of the hyomandibula. However, since oversegmentations can be easily corrected by our postprocessing tool, this is not problematic.

The plate-like tessera shape suggested the usage of a 2D approach but biological data consisting of different geometries might require another type of distance map. For example, Baum and Titschack [38] used an average distance for the segmentation of coral cavities in a μCT scan, since the cavities to be segmented were elongated rather than roundish or flat, and showed that the results were also superior to those from the 3D distance map.

When applying the **watershed segmentation**, there are different possibilities how to handle the often occurring oversegmentations. We used a hierarchical watershed scheme based on dynamics, in particular the size and depth of basins. This choice must be adjusted to the segmentation problem at hand. For example, Zanoguera et al. [39] used depth, area and volume dynamics for interactive segmentation.

The last step in our pipeline is the **postprocessing** based on the graph structure defined by the tesserae network. It is an optional step that can be omitted if the automatic segmentation results are sufficiently accurate. If necessary, a postprocessing tool should be tailored to correct the most common and most important errors in a fast and comfortable way.

## Conclusion

We have developed a semi-automatic segmentation pipeline to segment complex, biological tessellations in μCT data. Our pipeline allows successful segmentation of a single dataset in only a few hours, instead of days typically required for manual segmentation. The application of our pipeline is straightforward, and we provide tools to guide the easy determination of the necessary parameters. Our pipeline allows for semi-automatic segmentation of tiled flat objects by offering a new distance map that prevents many of the errors occurring in the commonly used 3D distance map and thereby greatly improves segmentation results. The remaining errors can be quickly and easily resolved with the proposed interactive tool, which makes use of a graph-based representation of the segmentation. Overall, our pipeline enables high-quality segmentations of complex volumetric (or image) data, of a type relevant to biological study, as demonstrated for the tesserae of whole skeletal elements. In the future, we will use our pipeline to characterize the tessellation of skeletal elements across multiple individuals and ages, creating a rich, quantitative perspective on how tiling varies inter-individually and across ontogeny (e.g. with respect to the morphometrics of tiles), that can then be related to more global properties of skeletal elements, like surface curvature. These and similar analyses of biological structure permitted by our pipeline open opportunities for flexible, quantitative exploration of large tomographic datasets, which are increasingly within reach through laboratory scanning facilities and open access data hosting.
